# The Inflammatory Cytokine Imbalance for Miscarriage, Pregnancy Loss and COVID-19 Pneumonia

**DOI:** 10.3389/fimmu.2022.861245

**Published:** 2022-03-11

**Authors:** Fortunato Vesce, Chiara Battisti, Michele Crudo

**Affiliations:** ^1^ OB & Gyn Complex Unit, Arcispedale Sant’Anna – Ferrara University, Ferrara, Italy; ^2^ Phytocal S.R.L., Rende (CS), Italy

**Keywords:** pregnancy inflammation, abortion, FIRS, SARS-COV-2, IL-6, viral pneumonia, cytokine storm

## Abstract

Pregnancy can be defined a vascular event upon endocrine control. In the human hemo-chorial placentation the chorionic villi penetrate the wall of the uterine spiral arteries, to provide increasing amounts of nutrients and oxygen for optimal fetal growth. In any physiological pregnancy the natural maternal response is of a Th1 inflammatory type, aimed at avoiding blood loss through the arteriolar wall openings. The control of the vascular function, during gestation as in any other condition, is achieved through the action of two main types of prostanoids: prostaglandin E2 and thromboxane on the one hand (for vasoconstriction and coagulation), prostacyclin on the other (for vasodilation and blood fluidification). The control of the maternal immune response is upon the responsibility of the fetus itself. Indeed, the chorionic villi are able to counteract the natural maternal response, thus changing the inflammatory Th1 type into the anti-inflammatory Th2. Clinical and experimental research in the past half century address to inflammation as the leading cause of abortion, pregnancy loss, premature delivery and related pulmonary, cerebral, intestinal fetal syndromes. Increased level of Interleukin 6, Interleukin 1-beta, Tumor Necrosis Factor-alfa, Interferon-gamma, are some among the well-known markers of gestational inflammation. On the other side, COVID-19 pneumonia is a result of extensive inflammation induced by viral replication within the cells of the respiratory tract. As it may happen in the uterine arteries in the absence of an effective fetal control, viral pneumonia triggers pulmonary vascular coagulation. The cytokines involved in the process are the same as those in gestational inflammation. As the fetus breathes throughout the placenta, fetal death from placental thrombosis is similar to adult death from pulmonary thrombosis. Preventing and counteracting inflammation is mandatory in both conditions. The most relevant literature dealing with the above-mentioned concepts is reviewed in the present article.

## Introduction

Despite the substantial progress in the comprehension of miscarriage and pregnancy loss pathogenic mechanism, there is still a great delay in transferring the scientific findings to medical practice. “Statistical evidence-based medicine” today is anxiously awaiting double blind randomized controlled trials, but forgets that these can only be planned by clinicians with a solid knowledge of pathophysiology. Science is measurement, but this must be based on a rational ground. In the absence of a solid philosophical perspective, the results of the measurements, however exact, end up supporting the wrong target. As a consequence, many obstetric complications leading to fetal demise are still generically attributed to a so called ‘placental insufficiency’, a definition sharply criticized long ago by H. Fox. This Author, indeed, pointed out that such complications are not a result of changes within the placenta, but of a compromised utero-placental blood flow. Furthermore, he stated that in these cases the placental maturation is accelerated, and therefore its functional efficiency is increased rather than reduced. This opinion is based on the finding of a decreased thickness of the placental barrier, with more precocious formation of vasculo-syncytial membranes, aimed at increasing the maternal-fetal passage of nutrients and gas exchange (1). At the time when Professor Fox described the morphological evidence of the vascular changes related to miscarriage and pregnancy loss, their inflammatory nature had not been yet understood. Indeed, in discussing the etiological classification of abortion, he observed for instance that trisomy 21 may result in a spectrum of abnormalities from blighted ovum to a mentally retarded adult, but the factors that allow the pregnancy survival are not known (1). As a matter of fact, although the pathogenic role of inflammation is better understood today, the criteria for a precise diagnosis and effective anti-inflammatory therapy have not been clearly expressed and are not adopted in clinical practice.

## Maternal Immune Tolerance and Susceptibility to Infection

Since the dawn of immunology, it was suggested that in normal pregnancy the maternal immune response must be reduced, to allow the acceptance of the semi-allogeneic product of conception.

Such a concept has created a myth of pregnancy as a state of immunological weakness and increased susceptibility to infectious diseases.

On the contrary, the immune gestational system, far from being suppressed, is modulated and perfectly working (2, 3).

The gestational decidua contains a high number of immune cells. Among leukocytes, 70% are NK cells, 20–25% are macrophages and 1.7% are dendritic cells (4, 5). During the first trimester, NK cells, dendritic cells and macrophages infiltrate the decidua and accumulate around the invading trophoblast cells (6, 7).

The decidual NK cells are critical for trophoblast invasion of the uterine spiral arteries, as their deletion leads to miscarriage (8, 9).

Contrary to the opinion of a decreased maternal immunity, there is also evidence for a boosted innate immune response implying a decreased susceptibility to infection, thus protecting the mother and the fetus (10, 11). Nevertheless, the reduction of the immune response, that could be leading to increased incidence of infection, suggested that pregnant women should be vaccinated against a list of diseases including flu, pertussis, tetanus, diphtheria, and more recently SARS-COV2 infection (12).

However, although the involvement of infectious agents is reported in about 40% of spontaneous abortion (13–15), there is controversy regarding the incidence and effects of infections on pregnancy. Indeed, the setting of pregnant women immunity would appear to be directed towards a better protection against infectious diseases compared to non-pregnant. For instance: vertical transmission of BKPyV, JCPyV and SV40 and specific IgG antibodies occurs in normal pregnancy (16); the incidence of Human Papilloma Virus (HPV) infection is not increased in spontaneous abortion. Moreover, the prevalence of serum anti-HPV16 IgG antibodies is 30% in normal pregnancy and 37.5% in women with spontaneous abortion (*p* > 0.05), thus suggesting even better humoral immunity in the latter (17); rubella virus, varicella-zoster, human immunodeficiency virus, adenovirus, cytomegalovirus, herpes simplex virus, human parvovirus, Epstein-Barr virus, enterovirus and respiratory syncytial virus, have all been found in amniotic fluid, but their mere presence appears not to be associated with negative human pregnancy outcome (18–21).

As for influenza, contrary to the opinion of some Authors (22–24), the risk for severe outcomes results to be decreased. Indeed, there is a significantly lower incidence of admission to intensive care unit. Moreover, neither a greater need for mechanical ventilatory support, nor a raised incidence of maternal death or other severe outcomes compared to general population are observed. As for the registered higher incidence of hospitalization, it is simply ascribed to a better care for motherhood (25).

## The Need to Distinguish Humoral From Cellular Gestational Immunity

All above results indicate that the opinion of an increased susceptibility to infectious disease in pregnancy may derive from purely statistical studies burdened by methodological errors, which make them unsuitable to settle disputes. These follow for the widespread confusion between the distinct functions of humoral and cellular immunity: the first, that is active in antibody production against infections, is perfectly preserved in pregnancy; the second, responsible for the acceptance of the fetus, is seriously challenged from implantation to delivery. There should be no need for references regarding the integrity of maternal antibody production: everyone knows, for instance, the effectiveness of humoral immunity in maternal-fetal Rh-immunization! In this case, the maternal humoral immune response is enhanced rather than decreased, i.e., the anti-Rh antibody production is triggered, with the specific intent of rejecting the semi-allogeneic fetus, instead of accepting it. Nevertheless, the maternal antibody response has been also tested against herpes simplex, measles, rubella and influenza A viruses, showing no consistent effect of pregnancy. It was stated that declining titers of antiviral antibodies may seldom be seen as a predominant effect of gestational physiological hemodilution (26).

Instead, changes are produced at the level of uterine vessels that maternal cellular immunity is not ready to accept. Two stages of such changes are described, the first beginning with implantation, and followed by a second wave in the second trimester (1). However, in our opinion, the process of uterine arteries invasion and progressive modification would better be considered to continue up to birth, due to the need of increasing amount of oxygen and nutrients for optimal fetal growth. A deep crosstalk between the invading trophoblast and the gestational decidua occurs. Trophoblast antigens stimulate all T-types cells, namely helper (Th), cytotoxic (Tc) and regulatory (Treg), as well as B cells, in order to induce a tightly regulated balance between inflammatory and tolerogenic states during the “immune chronology” of normal pregnancy (27–30). Naive CD3^+^CD4^+^ T cells differentiate into T cell subsets, such as Th1, Th2, Th9, Th17, Th22, and follicular Th cells (Tfh). Th1 inflammatory immunity dominates during implantation, in order to favor trophoblast invasion. Subsequently a shift to Th2 anti-inflammatory immunity is observed (31).

As regards the modulation of the inflammatory response, it must be considered that the mediators of cellular inflammation are also involved in the main physiological events of fertility, such as menstruation and delivery (32) Accordingly, the levels of the receptor ligands for the inflammatory peptide N-formyl-methionyl-leucyl-phenylalanine (fMLP) in amniotic fluid are stable during gestation, while they are significantly increased by labour, along with the expression of fMLP receptor in amnion tissue. These findings indicate that fMLP system modulates the events of physiological labour (33–35).

Ultimately, two aspects characterize the cellular immunity scenario that takes place at the maternal-foetal interface: the natural reaction to the penetration of the chorionic villi into the uterine spiral arteries on the one hand, and the foetal need to counteract it on the other. Indeed, the Th1 reaction, although probably worth to favour implantation, ends up later becoming a maternal defence against the vascular changes produced by the villi. Such a cellular inflammatory response is even more striking in the presence of maternal acute or chronic inflammatory disease, which already activate the cyclooxygenase pathways involved in prostanoids production.

At this regard, the complex relationship existing between endothelial cell receptors, prostanoids biosynthesis (36, 37) and cyclooxygenase influence on Th cells subset (38) must be considered. Based on the above evidence, it can be assumed that, from implantation to delivery, virtually all vascular events depend on the balance of inflammatory and anti-inflammatory cellular immune response, but not on the humoral ones. Moreover, regardless of its maternal or foetal origin, an excessive Th1 response causes inflammation and coagulation, thus leading to abortion and pregnancy loss.

## Role of the Th1 Immune Response in the Pathology of Gestation

As above mentioned, the trophoblast invasion generates a cellular immune response, the control of which is a task of the trophoblast itself, by releasing the right mediators of cellular functions needed in that unique condition of pregnancy generically called ‘maternal tolerance’ (39). Indeed, it is the trophoblast primary job to quench the Th1 reaction at the level of the utero-placental interface. To the best of our knowledge, Raghupathy was among the first to clearly affirm that Th1–type of immune response is incompatible with pregnancy (40). A more detailed description of the normal set-up of lymphocyte subsets mentioned above (31) suggests some benefit of Th1 immunity in the earliest stages of inflammation. Nevertheless, as Th1 cytokines are also able to trigger inflammation and coagulation, to confirm the hypothesis of a foetal responsibility in the control of these functions it was necessary to investigate upon their mediators in the foetal compartment of pregnancies at high risk of abortion. These are best represented by foetal aneuploidies. Accordingly, compared to euploid gestation, the following features were registered in the presence of chromosomal abnormalities:

- significantly increased amniotic fluid levels of endothelin-1 (41);
- significantly higher maternal serum levels of urokinase plasminogen activator and its complexed form with type-1 inhibitor.
- significantly lower amniotic fluid level of the tissue plasminogen activator in aneuploidy, with a higher amniotic level of type-1 inhibitor in the presence of minor chromosomal abnormalities (42);
- significantly increased level of amniotic fluid IL-6, with decreased IL-8 level, and reduced IL-6 concentration in the maternal blood (43);
- reduced adenosine receptors A (1) and A(2B) expression in chorionic villi and mesenchymal cells in the presence of fetal Trisomy (44)*;*

All above results strengthen the opinion of a leading pathogenic role of the fetal inflammatory cytokines, along with the vascular and blood clotting system anomalies, in the mechanism of miscarriage.

## Cytokine Imbalance in the Absence of Infection in Advanced Human Pregnancy Complications

As inflammation leading to miscarriage may derive from lack of fetal control of cytokines and prostanoids, one wonders if the same mechanism may be also involved in late pregnancy, when the time of abortion is now over.

At this regard it has been reported that the maintenance of Th1 immunity is linked to other late complications, such as fetal growth restriction, premature birth and related neonatal syndromes, gestosis, up to pregnancy loss (40). In the presence of fetal growth restriction, maternal peripheral mononuclear cells stimulation with trophoblast antigens produces higher levels of the pro-inflammatory cytokines IFNγ, TNFα, IL-8, IL-12, IL-18, IL-23 and lower anti-inflammatory cytokines IL-4, IL-10, IL-13 compared to normal fetal growth, thus confirming once more an active fetal role in the pathogenic mechanism (45, 46). Indeed, in such cases, the trophoblast even strengthens the maternal inflammatory response, instead of turning it off. Therefore, the question arises whether, and when, in the absence of aneuploidy, the persistence of Th1 immunity indicates an intrinsic inability of the euploid trophoblast, or it may recognize different etiologies, among which infection. Indeed, experimental intrauterine infection in primates triggers pro-inflammatory cytokine activation, prostaglandin release, myometrial contractions, and premature delivery (47).

As for human pregnancy, bacterial mediation would appear to be among the possible causes of a TH1 immune response. Indeed, in a recent article by Romero and coworkers, the finding of the same amniotic bacteria previously detected in the vagina at the time of amniocentesis suggested that pathogens ascension from the lower genital tract is the primary pathway for intra-amniotic infection leading to premature labor (48). In this research, IL-6 amniotic level was above 2 ng/ml, but it was not interpreted as an indicator of a preceding inflammation. However, the same Author, by sampling the fetal compartment in cases of threatened premature birth, had previously reported that fetal inflammation precedes infection (49, 50).

To the best of our knowledge, this is the first clinical demonstration that inflammation could not be triggered by infection. In humans, indeed, even the mere steril inflammation of the chorion-decidual interface is reported as a *primum movens* producing a cascade of cytokines that result in preterm birth (51). Accordingly, a recent article reports that T cell activation causes the following clinical signs of premature labor: maternal hypothermia, bradycardia, systemic inflammation, cervical dilation, intra-amniotic inflammation, and fetal growth restriction (52). Therefore, these findings confirm a leading role of inflammation in triggering premature birth and its related perinatal syndromes even in the absence of infection.

## Role of Fetal and Maternal Genetic Inflammatory Polymorphisms in the Pathogenic Mechanism of Gestational Inflammation and Its Related Clinical Complications

Further support for the predominant role of inflammation in the pathogenesis of complications of advanced pregnancy comes from the study of the involvement of genetic inflammatory polymorphisms. Indeed 119 genes with single nucleotide polymorphism are reported to be associated with preterm birth (53). It has been demonstrated that maternal polymorphisms in genes IL-10, MBL, TNFRSF6 and TGFB1 may influence susceptibility to chorioamnionitis (54). The risk of preterm birth is lower with polymorphisms decreasing the inflammatory response compared to those increasing its magnitude and or duration (55). Common genetic variants in proinflammatory cytokine genes IL-1α, IL-1ß, IL-2, IL-6, TNF, and lymphotoxin α, also increase the risk for spontaneous preterm birth (56). Interestingly, candidate gene studies have sought genetic variants regulating inflammation either in the mother or in the fetus. The most relevant concept derived from these studies is that preterm labor, at least in part, has an inflammatory etiology, that does not necessarily need to be triggered by pathogens: it is the so called ‘sterile intra-amniotic inflammation (57).

## Role of COVID-19 in Inflammation: The Cytokine Storm

Today the world is struggling from a global health emergency: the Coronavirus disease-19 (COVID-19), caused by SARS-CoV-2 virus infection. This infection triggers strong inflammatory responses leading to acute pneumonitis, bronchitis, dyspnea, and respiratory failure (58–62).

SARS-CoV-2 infection, called COVID-19, is often categorized into three stages: first asymptomatic phase; second, non-severe symptomatic phase; and third, severe respiratory symptomatic phase (63). Usually, a small number of patient’s progress to the severe stage and develop Acute Respiratory Distress Syndrome (ARDS) with or without multiorgan failure (64). In fact, the large part of people infected with SARS-CoV-2 occurs asymptomatically or cause only mild and less fatal symptoms than MERS-CoV and SARS-CoV infections. In 10–20% of cases, especially in those with associated comorbidities and advanced age, can progress to interstitial pneumonia and acute respiratory distress syndrome (ARDS) (65). Data suggest that the severity and high mortality rate of COVID-19 is related with older age with coexisting severely ills, nutritional status and serious comorbidity, especially pulmonary, cardiovascular and dysmetabolic ones, chronic obstructive lung disease and coronary heart disease. Otherwise, young and healthy people rarely developed severe COVID-19 pathology (66–71).

Studies have demonstrated that the host’s immune responses initiate as soon as SARS-CoV-2 binds to cellular receptors and releases viral RNA for replication with the involvement of both the innate and adaptive immune system (64). The immune response to the virus appeared to be different between severely and moderately COVID-19 patients (64). In a blood sample of symptomatic hospitalized patient with mild to moderate SARS-CoV-2 infection, before resolution of symptoms were detected immunological changes such as an increase in the number of activated CD4+ helper, T cells and CD8+ killer T cells, follicular helper T (Tfh) cells, antibody-secreting cells (ASCs) and antibodies particularly IgG (Immunoglobulin G) and IgM (Immunoglobulin M) (72). On the other hand, in severely infected patients, lymphocytopenia is a common denominator with substantial fall in numbers of natural killer cells, B cells, CD3+ T cells, CD4+ helper T cells, CD8+ killer T cells along with the increase in neutrophil-to-lymphocyte ratio (NLR) and C reactive protein levels (64). Additionally, in comparison to the non-severe patients, pro-inflammatory cytokines and chemokines such as tumor necrosis factor (TNF)-alpha, interleukin (IL)-2, IL-6, IL-7, IL-8, IL-10, Granulocyte-colony stimulating factor(GCSF), mono cytochemoattractant protein 1 (MCP1) and macrophage inflammatory protein 1-alpha (MIP1-alpha) are often reported to be elevated in serum levels of critically ill patients (66, 73). The elevated neutrophil-lymphocytes ratio (NLR), which is a biomarker of systemic inflammatory response syndrome, points to the devastated inflammatory state of COVID-19 patients in intensive care units (74). This hyperactive immune response along with impaired adaptive immune response may trigger pulmonary injury, ARDS, viral sepsis and organ failure as complications, and eventually death in some cases (74).

The severity of COVID-19 in patients is associated with an exaggerated immune response and intense inflammation due to a so called *“cytokine storm”*. Following COVID-19 infection, in the severe cases it was observed an excessive production of pro-inflammatory cytokines, namely tumor necrosis factor α (TNFα), interferon γ (INFγ), interleukin-6 (IL6), interleukin-1β (IL1β), and chemokines like monocyte chemoattractant protein-1 (MCP-1/CCL2). The immune-mediated cytokine storms are intended to protect the host from the infection; however, the excessive release of proinflammatory cytokines could harm multiple organs throughout the body.

The enveloped coronaviruses (CoVs) are a versatile family of positive-sense RNA viruses that infect several species and often in pleomorphic form (75). These are classified into four major categories according to their genomic structure as α, β, γ, and δ; the α and β CoVs affects only mammals. The SARS-CoV-2 and MERS-CoV-2 are grouped under β coronaviruses.

The CoVs have four structural proteins that includes E, M, N protein, plus S- Spike glycoprotein (76). Among these the S protein is located on the virion’s outer surface and it is the most crucial for the infection and its consequences on patients. The spike protein acts as a recognition factor as it attaches to the membrane receptor on the host cells, facilitating the fusion with cellular membrane (77).

The Spike protein mediates receptor recognition, cell attachment, and fusion during viral infection. The Spike protein are coated with polysaccharide molecules that camouflage them, allowing them to evade surveillance of the host immune system during entry (78). When the virus interacts with the host cell, there is an extensive structural rearrangement of the S protein that allows the virus to fuse with the host cell membrane and to penetrate into the cell (78).

In its native state, the CoVs S protein exists as an inactive precursor; however, during viral infection, specific cell proteases activate the S protein by cleaving it into S1 and S2 subunits (79); this step is necessary for activating the membrane fusion domain after viral entry into target cells (80). The S protein of SARS-CoV-2 is cleaved into S1 and S2 subunits by cellular proteases: S1 domain contains the Receptor binding domain (RBD), which is mainly responsible for binding of the virus to the receptor, while S2 domain mainly contains the heptad repeat (HR) domain, including HR1 and HR2, which is closely related to virus fusion (81). In the effort to explain the exaggerated immune responses observed in the severe COVID-19 patients, were performed structure-based computational models on S protein. By using in silico modeling, it was found that SARS-CoV-2 encodes a superantigen (SAg) region in SARS-CoV-2 S glycoprotein that is highly similar in sequence and structure to the staphylococcal enterotoxins B (SEB) (82). Bacterial SAgs, like SEB, include proteins that stimulate massive production of inflammatory cytokines and toxic shock. SAgs are able bind to major histocompatibility complex (MHC) class II (MHCII) molecules and/or to T cell receptors (TCRs) of both CD4+ and CD8+ T cells, and they are well known as potent T cell activators (82). The SAgs have the capacity to bypass the antigen specificity of the TCRs and to cause a broad activation of T cells that lead to a cytokine storm and to toxic shock (83, 84). SAgs do not bind the major (antigenic) peptide-binding groove of MHCII but, instead, directly other regions of MHCII, and recent studies revealed that they can bind to either α- or β-chains or both the TCRs (85). SEB enables large-scale T cell activation and proliferation with a massive production of proinflammatory cytokines including IFNγ, TNFα, and IL-2 from T cells, as well as IL-1 and TNFα from antigen-presenting cells, that finally leads to multiorgan tissue damage (84).

The hyper-inflammatory syndrome, observed in severe cases of COVID-19 in adults, may be driven by the SAg-like activity of the S protein (82). Indeed, the inflammatory cytokine signature, that include IL-6, TNFα, IL-8, and IL-1β, and which predicts severity and possible death in COVID-19 patients, is very similar to the one elicited by SAgs (84, 86). It was also noted that adult patients with severe/hyper-inflammatory COVID-19 exhibit a skewed TCR Vβ repertoire, similar to the one elicited by bacterial SAgs, that distinguish them from patients with mild/moderate COVID-19 (82). A discriminant in the severity of COVID-19 symptoms in patients could also be related to the HLA systems. HLA has been shown to play a role in COVID-19 susceptibility, and it is known that certain HLA types are more permissive of binding SAg (87). SAgs have been also implicated in autoimmunity by triggering self-reactive T cells (83). In a similar way, it is also possible that SARS-CoV-2 SAg could cause a delayed hyper-inflammatory response by antibody-mediated enhancement due to the virus re-exposure (88) when a poor initial antibody response to the virus fails to neutralize the SAg (82).

## Effects of SARS-CoV-2 S Protein on ACE-2 in Pregnancy

Aside respiratory problems and the S protein-induced cytokine storm, the SARS-CoV-2 virus targets cardiac, gastro-intestinal, hepatic, cutaneous, and renal organs to elicit organopathy. This is possible because SARS-CoV-2 virus uses angiotensin converting enzyme 2 (ACE-2), trans-membrane serine protease 2 and 4 (TMPRSS2, TMPRSS4), glucose regulating protein 78 (GRP78) Cluster of Differentiation 147 (CD147), and other entry factors to colonize host cells (89). The organs expressing these receptors are at greater risk to show COVID-19 organ dysfunctions. In this contest, it is important to underline that several studies have showed the expression of these entry factors in gonads and other accessory reproductive organs (59–61). In fact, especially ACE-2 is largely expressed in the reproductive system, ovary (especially, in a high rate, in oocytes) (90–92), utherus (92–94) and vagina (92, 94) included. The role of ACE2 and, in particularly, the modulation of the concentration of Angiotensin II and Angiotensin- (1–7), is fundamental for the correct function of menstrual cycle and fertility of woman. ACE2 is able to hydrolyze angiotensin I (Ang I) to produce angiotensin- (1–9) and also has a high affinity for angiotensin II (Ang II) to generate Ang-(1-7) (95). The major component of the ACE/Ang II/AT1 (angiotensin II type 1) axis, is Angiotensin II, that maintains the hydro-salinity balance (96, 97) and promotes cell proliferation and facilitates vasoconstriction (98–101). Ang-(1-7). is an endogenous ligand for the G protein-coupled receptor Mas (102, 103) and, as an important modulator of the human renin-angiotensin system (RAS), specifically inhibits Ang II by the antagonism of AT1 receptors (103). Moreover, Ang-(1-7) alleviates metabolic syndrome (104, 105), enhances vasodilation (106, 107) and seems to protect the heart (108–110). Several studies have demonstrated that ACE2 is a modulator of the secretion of Angiotensin II and Ang (–1–7). The natural balance of this two molecules is associated with maturation of human oocytes (111–114), induction of hormones secretion (115–117), follicle development (114, 116, 118–122) and atresia (123–125), and modulation of the ovulation (114, 126–136). Furthermore, a correct balance between Ang II and Ang-(1-7) plays an important role in vascular bed and endometrium regeneration, and it is necessary for a regular menstrual cycle (137, 138), for the regeneration of endometrium (93) and myometrium activity (139, 140).

In addition, the human placental RAS, that is directly modulated from ACE-2, is upregulated in the first trimester and involved in endometrial neo-vascularization during peri-implantation period as well as placental development period (141).

During pregnancy, the balance between Angiotensin II and Ang-(1-7) is fundamental and ACE-2 is widely expressed in human cytotrophoblast, syncytiotrophoblast, endothelium and vascular smooth muscle of the chorionic villi (142). The presence of ACE-2 is detected even in the maternal stroma, in the invading and intravascular trophoblast, in decidual cells, in arterial and venous endothelium and smooth muscle of the umbilical cord (142). ACE-2 reaches the highest level in early gestation (143), where it is expressed in luminal and glandular epithelial cells in the primary and secondary decidual zone. During gestation, ACE-2 staining is visualized in the placenta, and amniotic and yolk sac epithelium (144, 145). ACE-2, Ang II and Ang-(1-7), seem to be involved in the regulation of blood pressure, hydro-salinity balance fetus development (143). In particularly, Ang II seems to stimulate trophoblast invasion in human cells (146), while Ang-(1-7) and ACE2 may behave as a local autocrine/paracrine regulator in the early (angiogenesis, apoptosis and growth) and late (uteroplacental blood flow) events of pregnancy (144). Several studies suggested that an abnormal expression of Ang II, ACE-2 and Ang-(1-7) may induce pre-eclampsia (147–152). In fact, during pre-eclampsia, high expression of Ang II in the placental villi causes a decreased blood flow and fetal nutrition (149, 150, 153). Low placenta levels of ACE-2 and Ang-(1-7) are instead associated with intrauterine growth restriction (145).

Dysregulation of RAS has been even linked with miscarriage reported (140).

A recent study showed that in pregnant SARS-CoV-2 patients, ACE-2 levels were lower in asymptomatic/mild patients compared to those with severe disease (154). It was observed that during SARS-CoV-2 infection, the binding of S1 to the ACE2 receptor induce the cleavage of ACE2 by a disintegrin and metallopeptidase domain 17 (ADAM17)/tumor necrosis factor-converting enzyme (TACE) at the ectodomain sites (155–157). Furthermore, TMPRSS2 cleaves ACE2 at the intracellular C-terminal domain (80, 156). These processes lead to shedding of host ACE2 receptor (158) that may contribute to the loss of ACE-2 function (159). In this way, SARS-CoV-2-ACE2 complex negatively regulates ACE-2, leading to a decrease in plasma levels of angiotensin-(1–7), potentiating vasoconstriction and hyper-coagulation and therefore contributing to reproductive failure and other obstetrical complications (160).

Moreover, SARS-CoV-2 infection during the preconception period and the first half of pregnancy may increase the risk of miscarriage by affecting the ACE-2 activity (161). It has been *in vitro* shown that S1 subunit of SARS-CoV-2 S protein without the rest of the viral component was alone able to induce ACE-2 mediated cell signaling (71, 162, 163).

Spontaneous miscarriage and preterm delivery have been documented among coronavirus infected pregnant women in the past (62). At the immuno-pathological levels, the reproductive failure is a consequence of a pro-inflammatory maternal immune response, thromboembolic events, or it could be a direct consequence of the virus activity in the uterine environment (endometrium, decidua, and trophoblast). Therefore, SARS-CoV-2 may affect the reproductive health by inducing cytokines storm in infected pregnant women (164), as well as by the direct action of S1 subunits of S protein on ACE-2 expressing cells in the reproductive system (71).

## Some Basic Principles for Anti-Inflammatory Therapeutic Approach: Lactoferrin, Antibiotics, Glucocorticoids, Tocilizumab, α-1-Antitrypsin

Despite the previous experience with SARS and MERS, health systems around the world were not prepared to fight against the COVID-19 pandemic. Although the new virus shares a large part of its structure with the preceding coronaviruses, triggering a clinical form of pneumonia quite similar to all others, WHO suggestions led to not treating the initial presentation of the disease, pending the production of vaccines. Therefore, the majority of patients were hospitalized when ventilatory support was now useless due to pulmonary thrombosis, and medical therapies were no longer able to extinguish inflammation and superimposed infection. However, vaccination being a preventive measure, and prevention a different topic from therapy, one might be wondering if there is a rational therapeutic approach for gestational and viral inflammation, which, although different in etiology, share the same pathogenesis.

In general, it can be argued that some predisposing factors for inflammation or clotting, such as genetics, cannot be modified. Their consequences, instead, may be mitigated, even by administration of simple supplements. One of them is Lactoferrin (LF), an iron-binding glycoprotein largely used to cure anemia. In fact, a significant decrease of IL-6 amniotic concentration has been registered 4 hours after 300 mg transvaginal LF intake (165). The same dose down-regulates 17 pro-inflammatory amniotic cytokines among which IL-9, IL-15, IFN-γ, IP-10, TNF-α, IL-1α and MCP-3, while up-regulating several among anti-inflammatory (166). Moreover, LF also lowers PGE 2, active MMP-9, and its inhibitor TIMP-1, while increasing active MMP-2 and MMP-2/TIMP-2 molar ratio, and leaving unchanged TIMP-2 (167). To look for a single drug capable of balancing the intricate network of stormy cytokines is a legitimate but naive hope: lowering the level of just one cytokine while that of many others remains high doesn’t make much sense. Therefore the attention of researchers should turn to drugs capable of restoring the balance of cytokines as a whole, reducing the level of the inflammatory ones and increasing that of the anti-inflammatory, as happens with lactoferrin and cortisone (168).

Infact, the physiological defense against inflammation is based on the production of glucocorticoids by the adrenal gland. Their circadian release regulates the mediators of cellular functions among which IL-1, IL-6, IL-8, Tumor necrosis factor, granulocyte-macrophage colony-stimulating factor (G-CSF), and monocyte chemotactic protein-1 (MCP-1) (169). Glucocorticoids also regulate the cellular cytokine receptors (170, 171). Indeed, the expression of the receptors that recognize a variety of pathogens (Toll-like), is down-regulated (172), that of pro-inflammatory cytokines is suppressed, and that of anti-inflammatory is up-regulated by dexamethasone in isolated murine liver cells (173).

In addition, GCs inhibit the human pro-IL-lβ gene by decreasing DNA binding of trans activators to the signal-responsive enhancer (174).

It is also worth to note that the glucocorticoid receptor exerts autonomous control of TNF stimulated IL6 release by decreasing it independently from GCs (175).

A further example of complexity of the glucocorticoid system regulation is the hormone induction of MIF secretion (176), thereby apparently counteracting its anti-inflammatory mission.

Several other aspects need to be considered in the prevention and therapy of the devastating effects of gestational inflammation. First, the action of cortisol, the hormonal form that is active in the body, is regulated at the cellular level by 11-beta-Hydroxysteroid-Dehydrogenase, the enzyme that transform it into cortisone, its inactive form. The activity of the enzyme is high in the chorionic villi, while it is lacking in the embryo in the early stages of development, thus implying that nature does not fear the action of the hormone during the most delicate phase of morphogenesis (177). Glucocorticoids with different anti-inflammatory power are available (178) and it is quite common in obstetrics to resort to drugs with low anti-inflammatory power, for fears of causing harm to the fetus. This would appear the main reason why the Guidelines recommend prednisolone instead of the much more potent betamethasone or dexamethasone. But once the drug reaches the level of the chorionic villi, that is exactly where its action is required, its weak anti-inflammatory power is completely eliminated by the enzyme. A clinical confirmation can be found in the case reported by Queenby of a patient with history of 19 consecutive miscarriages (179). After losing the first14 pregnancies, due to the finding of several uterine natural killer cells considered too high, the lady was treated with prednisolone 5 mg daily, bringing to 19 the total number of miscarriages. Finally, the daily dose of the drug was increased to 20 mg, and she became able to reach the eighth month of pregnancy, when she gave birth to a healthy baby. Instead, since the fluorine prevents the action of 11-beta-Hydroxysteroid-Dehydrogenase, the fluorinated glucocorticoids, such as betamethasone, are endowed with much greater anti-inflammatory power, which they keep intact at the chorionic level, therefore resulting fully effective at the level of utero-placental interface. This synthetic glucocorticoid, neglected in early gestational age, is widely used instead in advanced pregnancy for preventing the Hyaline Membrane Disease of the premature neonate. Introduced half a century ago (180), this therapy is still believed to induce a sort of so called *‘maturation’* of type II alveolar cells, thus increasing their production of pulmonary surfactant. Two evidences are reported in support of the concept of *‘pulmonary maturation’* following betamethasone administration: the increased amniotic concentration of lecithin, and the decreased incidence of the respiratory distress syndrome (RDS) of the premature baby. However, it has been shown that betamethasone induces the release of a large amount of lecithin from amnion (181). Moreover, the reduced incidence of neonatal RDS, along with that of cerebral and intestinal damage, is a result of the anti-inflammatory power of the hormone. Therefore, since the syndromes of the premature baby (Hyaline Membrane Disease, Perinatal Encephalopathy and Neonatal Enterocolitis) are not due to prematurity by itself, but to inflammation leading to premature birth, it is wrong and misleading to maintain that betamethasone improves *‘maturation’*, instead of pointing out that it turns off inflammation. As inflammation begins days or weeks before symptoms appear, one can hypothesize that, in cases at risk, an earlier cortisone therapy at the appropriate doses, can prevent premature labor (182, 183).

Although the efficacy of the therapy largely depends on the stage of cytokine imbalance syndromes, there are cases of extreme severity of the foetal-maternal inflammation where glucocorticoids are not able to revert the process to normal anymore. The same happens in the most advanced stage of COVID-19 infection, and in the ‘Secondary hemo-phagocytic lymphohistiocytosis’ (sHLH), a syndrome that can follow chimeric antigen receptor T cell (CAR) therapy for acute lymphoblastic leukaemia. Furthermore, it has been hypothesized that glucocorticoids may delay viral clearance (184, 185). For such reason the WHO suggest to avoid their use for treating COVID-19 infection.

Attempts have been made to reduce the level of IL-6 by administering its antagonist Tocilizumab (186). Tocilizumab, the receptor antagonist IL-6R, was given intravenously, with rapid disappearance of fever respiratory symptoms and hypotension (187). This monoclonal antibody Immunoglobulin G (IgG) has been used also during pregnancy. It does not cross the placental barrier during the first trimester, although its passage becomes maximum in the third trimester, with an increased risk of preterm labor (188, 189). It seems unlikely that tocilizumab alone would be able to rebalance all cytokines and prostanoids affected by gestational or viral inflammation. However, its use could be of great value when untreated inflammation reaches the extreme stage of cytokine storm.

α-1-antitrypsin (AAT), another anti-inflammatory medication that is recently suggested to use, is a serine protease inhibitor providing a defense against the digestion of healthy tissue by proteolytic enzymes. Interestingly, AAT blood level is very high during inflammation, as well as in advanced pregnancy, while its deficiency causes inflammation and viral infections. AAT therapy has been approved for treatment of chronic obstructive pulmonary disease (190), and there is no reason not to test it, even as a preventive measure, in a serious emergency as that of the current pandemic.

A procedure that proved effective in saving many human lives is hyperimmune serum transfusions from recovered patients (191). Their efficacy depends on a direct neutralization of the virus, by preventing its entry into the cell.

## Concluding Remarks

Fetal and maternal death in pregnancy, and death from COVID-19 infection share the same pathogenic mechanism. Indeed, COVID-19 inflammation triggered by unbalanced cytokines, followed by coagulation, takes place in the lungs, while in pregnant women the same occurs at the utero-placental level. In COVID-19 the cause of the inflammation is the virus, while in pregnancy, as reported above, it is the fetus itself: therefore, in this latter case the cause cannot be eliminated. Nevertheless, the cure should be the same in both conditions. The rationale for management does not consists in fighting the cause, but in curing the disease, i.e. inflammation, and consequent overlapping bacterial infection and thrombosis. Therapeutic agents include cortisone and eventually other non-steroidal drugs against inflammation, as well as heparin to treat thrombosis. However it can be stated: no cytokine imbalance=no inflammation, no inflammation=no intravascular coagulation. Moreover, it must be stressed that the treatment is all the more effective the earlier it is started. The same therapy that can be effective if started at the first onset of symptoms, becomes compassionate if started when inflammation and thrombosis are already at an advanced stage.

## Author Contributions

FV projected the study and is responsible for clinical implications. CB and MC equally contributed to pregnancy loss and COVID-19 literature review. All authors contributed to the article and approved the submitted version.

## Conflict of Interest

Authors Chiara Battisti and Michele Crudo were employed by the company Phytocal SRL.

The remaining author declare that the research was conducted in the absence of any commercial or financial relationships that could be construed as a potential conflict of interest.

There are no grants or commercial funding.

## Publisher’s Note

All claims expressed in this article are solely those of the authors and do not necessarily represent those of their affiliated organizations, or those of the publisher, the editors and the reviewers. Any product that may be evaluated in this article, or claim that may be made by its manufacturer, is not guaranteed or endorsed by the publisher.
